# Press Comments on the Death of Dr. Taft

**Published:** 1903-11-15

**Authors:** 


### PRESS COMMENTS ON THE DEATH OF DR. TAFT.

### Dr. W. F. Litch, in *Dental Brief.*

The intelligence that Dr. Jonathan Taft has passed from the scene of his earthly activities will be received with profound regret by the hosts of friends who knew his sterling worth and appreciated his loyalty and devotion to the best interests of the profession to whose service his long and useful life was devoted.

Perhaps the feature of his character which was most "express and admirable" was the impeccable soundness of his moral fiber. Crookedness and indirection were foreign to his nature, and to all the activities of his long, honorable, and useful career he brought sterling integrity and absolute loyalty to truth.

Uniformly urbane and courteous in social intercourse, and in the highest sense a gentleman, when aroused he had at command large potencies of incisive speech with which to enforce a lesson, expose a fallacy, or condemn a wrong.

A veteran in dentistry and a pioneer in all those progressive advances which finally culminated in its organization and recognition as a distinct professional body, he has finished his work.

DR. J. N. CROUSE, IN *Dental Digest.*

We are called upon to record the death of Dr. Jonathan Taft, one of the pioneers of our profession and one who has done as much if not more for the upbuilding and advancement of dentistry than any other one man. His service to his profession was not alone in educational and literary work, but also in the influence of his personal character. A man of strong will power, he was nevertheless always ready to make concessions and defer to the wishes and judgment of others, where principle was not involved. Although lame and forced to use a cane nearly all his life, and never strong nor in good health, he was ever cheerful and full of sympathy and encouragement for others. His genial greeting and hearty handshake were a benediction. No one could come in contact with him without being benefited. His friends were legion and his enemies few, if any. He was well called the Grand Old Man of Dentistry.

The giants of our profession are fast dropping out, and the question arises, will their places be filled by the present or future generations of dentists? The men entering practice to-day are perhaps better educated, and they certainly have better opportunities for perfecting themselves in all branches. The danger is, however, especially in this age of commercialism, that they will lose sight of the ideals toward which they should struggle, because things are made so easy for them. The men of to-day are quite as strong morally and intellectually as those of any previous generation, but the same opportunities for development of character are not present. In the early days the struggle necessary not only for advancement and knowledge, but for a bare existence, of necessity developed the dental practitioner, but to-day his path is made easy, and if he is to attain the heights the pioneers reached he must be something more than a merely successful and capable operator. No young man to-day has the same opportunity for pioneer work as had Dr. Taft, but each can emulate his character, and strive in his own way to do his full duty by himself, his profession and his patients.

### Dr. Jas. Truman, in *International Journal.*

The life of such a man can scarcely be written by his contemporaries with justice to his memory. It has been too full of activities, in a professional sense, for more than a half-century, to be compassed in a few lines. Men die and their works live after them to enlighten and encourage those who have attempted to follow in their footsteps.

Dr. Taft occupied a position in dentistry, if not original, at least unique in its character. He was not a devotee of what is termed scientific investigation; indeed, it may be doubted whether he was original, as that word is understood, yet he obsorbed the work of other men so thoroughly that he became known throughout the country as an educator, with few, if any equals. It was from this standard that his work must be judged.

Dr. Taft was one not to enter into warm disputations. His life was too serene, and his religious nature avoided the tempestuous conflicts that frequently disturbed the annual dental gatherings. He doubtless realized that storms and tempests are transitory and would end with a serener sky, and for this he could patiently wait. He was, therefore, never a partisan.

During the entire life of the American Dental Association he was a recognized authority. As far as the writer is aware, he never missed the annual gatherings of this body, no matter how far the meeting might be from his home.

He was one of the original organizers of the National Association of Dental Faculties, and has participated in its work up to the present year. He took an early and active interest in dental education, and organized the Dental Department of Michigan University.

His nature was lovable and earnest. His whole life was devoted to his profession and his religious duties, and this he has left to those who still work in the ranks as a precious heritage. If his true professional spirit could enter into the life of dentistry to-day, how much better it would be for all who claim to be members of it as a profession. He belonged to a class of whom, unfortunately, we have too few, and the few are not appreciated.

While Dr. Taft had reached the limit of age ordinarily given to man, and while we cannot mourn his departure as we would the young, yet the world, and especially the dental world, has reason to regret that such as he cannot remain a blessing and an example to those most in need of such a gracious life. He was a warm friend, and as such the writer will miss him as the days go on, but with unfailing trust in the future that as men die men will live, and in the future the world will come to realize that death and life embody the idea of eternal progress.

Dr. C. N. Pierce, of Philadelphia, in *Dental Brief.*

My personal acquaintance with Dr. Taft began forty-five years ago, when, accompanied by Dr. George Watt, he visited Philadelphia on behalf of some one of those professional interests whose promotion was then, as throughout his entire professional career, one of the chief objects of his life.

During that visit I for the first time met him, and since then have been intimately associated with him in educational work. Thus nearly two generations have passed away since our friendship was sealed by a common interest.

After a life extending much beyond the allotted span, throughout which he maintained comparative health and professional activity, he has gone over to the majority, leaving a name as educator, author, and practitioner second to none in the records of the profession he loved and adorned.

Fully appreciating the advantages of an interchange of thought, he was always active in dental association meetings, and when addressing the assembly he did it with a force and continuity of thought which insured respectful attention even from those to whom his arguments did not carry conviction.

On the organization of the American Dental Association at Niagara Falls in August, 1859, he was chosen its first secretary, this being the preliminary meeting, and was re-elected to that position at the regular meeting held in Washington July 31, 1860. In 1869 he was elected president. In addition to his duties as secretary that year he read an essay on the buccal secretions, explaining the object of this fluid as being mechanical, chemical and dynamical. In the same paper some space was devoted to the pathological conditions which were indicated by the acidity or alkalinity of the saliva, and, as the latter quality predominated, how salivary calculus was rapidly deposited upon the teeth. In addition to these, other modifications and influences of the saliva were noted. Of this paper it may be said that it might have been written to-day instead of fifty years ago, so little is it at variance with the results of modern investigation.

Dr. Taft each year prepared reports for the American Dental Association; on several occasions dental literature and nomenclature were the subjects discussed. These contributions were always clear and instructive, demonstrating that the writer was fully conversant with the subjects considered.

Upon whatever subject he was writing or speaking he had decided views, and usually expressed himself in sympathy with all improvements on traditional customs, especially in teaching methods. His essays on professional education were marked by a thoroughly broad yet conservative spirit.

While Dr. Taft rarely contributed to those branches of dental science dealing with tooth development, embryology, or histology, yet he was always interested in that field of work and was in full sympathy with those who contributed to its literature.

As early as 1874 he wrote on dental education, taking the position that great imperfection existed in the instruction of dental students, and that much more might be done than was accomplished by the course of instruction then generally pursued. This he claimed was true not only of private or office instruction, but of that given in schools. Regarding colleges as the chief factors in determining the standard of the profession, and in order that that standard might be raised, he early declared himself in favor of a studentship of three years, and as opposed to the granting of honorary degrees in any case except as a recognition of eminent professional service.

